# Next-generation multimodality of nutrigenomic cancer therapy: sulforaphane in combination with acetazolamide actively target bronchial carcinoid cancer in disabling the PI3K/Akt/mTOR survival pathway and inducing apoptosis

**DOI:** 10.18632/oncotarget.28011

**Published:** 2021-07-20

**Authors:** Reza Bayat Mokhtari, Bessi Qorri, Narges Baluch, Angelo Sparaneo, Federico Pio Fabrizio, Lucia Anna Muscarella, Albina Tyker, Sushil Kumar, Hai-Ling Margaret Cheng, Myron R. Szewczuk, Bikul Das, Herman Yeger

**Affiliations:** ^1^Program in Developmental and Stem Cell Biology, The Hospital for Sick Children, Toronto, Ontario, Canada; ^2^Department of Experimental Therapeutics, Thoreau Laboratory for Global Health, M2D2, University of Massachusetts, Lowell, MA, USA; ^3^Department of Biomedical and Molecular Sciences, Queen’s University, Kingston, Ontario, Canada; ^4^Department of Immunology and Allergy, The Hospital for Sick Children, Toronto, Ontario, Canada; ^5^Laboratory of Oncology, IRCCS Casa Sollievo della Sofferenza, San Giovanni Rotondo FG, Italy; ^6^Department of Internal Medicine, University of Chicago, Chicago, IL, USA; ^7^Q.P.S. Holdings LLC, Pencader Corporate Center, Newark, DE, USA; ^8^Institute of Biomedical Engineering, The Edward S. Rogers Sr. Department of Electrical & Computer Engineering, University of Toronto, Toronto, Canada; ^9^Department of Cancer and Stem Cell Biology, KaviKrishna Laboratory, Guwahati Biotech Park, Indian Institute of Technology, Guwahati, Assam, India; ^10^Department of Immunology and Infectious Diseases, Forsyth Institute, Cambridge, MA, USA

**Keywords:** sulforaphane, acetazolamide, bronchial carcinoid tumors, serotonin, carbonic anhydrase

## Abstract

Objective: Aberrations in the PI3K/AKT/mTOR survival pathway in many cancers are the most common genomic abnormalities. The phytochemical and bioactive agent sulforaphane (SFN) has nutrigenomic potential in activating the expression of several cellular protective genes via the transcription factor nuclear factor erythroid 2-related factor 2 (Nrf2). Nrf2 is primarily related to mechanisms of endogenous cellular defense and survival. The efficacy of SFN in combination with acetazolamide (AZ) was investigated in reducing typical H727 and atypical H720 BC survival, migration potential, and apoptosis *in vitro* and *in vivo* preclinical xenograft tissues.

Materials and Methods: Microscopic imaging, immunocytochemistry, wound healing assay, caspase-cleaved cytokeratin 18 (M30, CCK18) CytoDeath ELISA assay, immunofluorescence labeling assays for apoptosis, hypoxia, Western Blotting, Tunnel assay, measurement of 5-HT secretion by carbon fiber amperometry assay, quantitative methylation-specific PCR (qMSP), morphologic changes, cell viability, apoptosis activity and the expression levels of phospho-Akt1, Akt1, HIF-1α, PI3K, p21, CAIX, 5-HT, phospho-mTOR, and mTOR in xenografts derived from typical H727 and atypical H720 BC cell lines.

Results: Combining AZ+SFN reduced tumor cell survival compared to each agent alone, both *in vitro* and *in vivo* xenograft tissues. AZ+SFN targeted multiple pathways involved in cell cycle, serotonin secretion, survival, and growth pathways, highlighting its therapeutic approach. Both H727 and H720 cells were associated with induction of apoptosis, upregulation of the p21 cell cycle inhibitor, and downregulation of the PI3K/Akt/mTOR pathway, suggesting that the PI3K/Akt/mTOR pathway is a primary target of the AZ+SFN combination therapy.

Conclusions: Combining SFN+AZ significantly inhibits the PI3K/Akt/mTOR pathway and significantly reducing 5-HT secretion in carcinoid syndrome.

## INTRODUCTION

Malignant cells are characterized by the upregulation and activation of many survival signaling pathways involved in proliferation, apoptosis, invasion, and angiogenesis [1]. In malignancy, many proteins and signaling pathways are observed to be upregulated in opposing cells’ malignant behavior. For example, neuroendocrine tumors (NETs) are malignant neoplasms derived from the neuroendocrine cells of primitive foregut, midgut, and hindgut structures. In the lung, these neoplasms are classified into high-grade carcinomas and low-grade bronchial carcinoids (BCs). Overexpression of hypoxia-inducible factor (HIF-1α), histone deacetylase (HDAC), and carbonic anhydrase IX (CAIX), as well as constitutive activation of the Akt/NF-kB pathway, have been implicated in carcinoid tumorigenesis [2–4]. The phosphatidylinositol 3-Kinase-Akt (PI3K-Akt) pathway is one of the critical cancer pathogenic pathways with widespread downstream effects involving cell cycle survival and hypoxic metabolic response, angiogenesis, and metastasis [5]. The PI3K-Akt pathway is highly active in BC tumors, promoting proliferation, growth, and inhibition of apoptosis via activation of DNA repair mechanisms [6, 7]. Further downstream activation of the Akt/NF-kB pathway can inactivate apoptotic pathways and promote the transcription of numerous pro-survival genes, including those responsible for angiogenesis [8]. As the PI3K-Akt pathway is overactive in many cancers, including non-small cell lung cancer, it is another potential therapeutic target in BC [9].

HIF-1α is a well-described mediator of the hypoxic response in carcinoid tumors, responsible for regulating genes critical for tumor cell survival in low oxygen conditions, including CAIX and nuclear factor kappa B transcription factor (NF-κB) [10]. In ileal carcinoids, HIF-1α is constitutively active and associated with gene expression related to epithelial-to-mesenchymal transition (EMT) and tumor cell migration [11]. Carbonic anhydrases (CAs) are critical regulators of cellular pH by converting carbon dioxide to bicarbonate. Transcription of CAIX, a membrane-associated CA, is chiefly regulated by the HIF-1α family resulting in an acidic extracellular environment that promotes the expansion of the cancer stem cell (CSC) population, invasion, and tumor metastasis [10, 12]. Interestingly, human embryonic stem cells (hESCs) exposed to an extreme environment of hypoxia and oxidative stress exhibited high HIF-2α and low p53 activity induced a transient state of reprogramming to a higher state of enhanced stemness having very high Nanog expression, high antioxidant secretion, and high cytoprotective activity. [13] We have reported that sulforaphane (SFN), acetazolamide (AZ), and AZ+SFN reduces the expression of stem cell markers (ALDH1, CD44, OCT4, SOX2, and Nanog) in BC cells [14]. A reduction in the CSC population may be one of the mechanisms by which AZ+SFN reduced the tumorigenicity of the H727 and H720 cell lines. As a result, CAIX transcription has been identified as a core pro-survival mechanism and a marker of poor prognosis and tumor hypoxia in many cancer types, including renal cell carcinoma and BC [3, 15]. A second mechanism promoting tumor cell survival in hypoxic conditions is the nuclear factor (erythroid-derived 2)-like 2-kelch-like ECH-associated protein 1 (Keap1-Nrf2) pathway [16]. This pathway regulates the tumor microenvironment and promotes tumor cell survival by reducing reactive oxygen species (ROS) and subsequent DNA damage within tumor cells [16]. Lung, ovarian, gallbladder, and liver cancers are known to have defects in Keap1, leading to Nrf2 over-activation [17]. Thus, targeting the Keap1-Nrf2 pathway may have widespread anti-tumorigenic effects.

Collectively, targeted multimodal approaches need to be strategically developed to take advantage of multiple pro-survival pathways, including tumor cell evasion, ROS, apoptosis, metastasis, and hypoxia. In many cancers, aberrations in the PI3K/Akt/mTOR survival pathway are the most common genomic abnormalities. Here, the next-generation multimodality using nutrigenomic therapy as an alternate approach with natural bioactive chemical interventions is likely to prevent cancer growth [1, 18]. The phytochemical and bioactive SFN has nutrigenomic potential in activating the expression of several cellular protective genes via the transcription factor Nrf2, primarily related to mechanisms of endogenous cellular defense and survival. We have previously reported that SFN in combination with AZ, a pan-carbonic anhydrase inhibitor, significantly inhibited the viability, clonogenicity, and *in vitro* growth of H727 (typical carcinoid) and H720 (atypical carcinoid) BC cell lines [3]. Also, using an orthotopic lung model of bronchial carcinoid, cell line-derived spheroids, and patient tumor-derived 3rd generation spheroids under supplemental stroma media conditions, we reported that SFN in combination with AZ significantly inhibited the growth of the BC cell lines (H727 and H720), the formation of spheroids containing a higher fraction of tumor-initiating cells (TIC) exhibiting a stemness phenotype, and in reducing tumor formation in immunocompromised mice [14].

In this study, we investigated the mechanism(s) by which SFN combined with AZ exerts its nutrigenetic therapeutic effect on BC cell lines and in BC xenograft tissues derived from H727 and H720 BC cells previously developed in NOD/SCID mice. The combination of SFN and AZ reduced the pro-survival PI3K/Akt/mTOR pathway, upended pro-survival hypoxia-mediated pathways resulting in decreased 5-HT secretion, migration of H727 and H720 cells in xenografts, and targeted the pro-survival Keap1/Nrf2 pathway, with an overall marked induction of BC cell apoptosis. For the first time, these findings demonstrate that SFN in combination with AZ provides a practical nutrigenetic therapeutic approach in targeting multistage pro-survival pathways in bronchial carcinoids.

## RESULTS

### AZ, SFN, and the combination of AZ+SFN reduce scratch wound closure of H727 typical carcinoid cells

**Figure 1:**
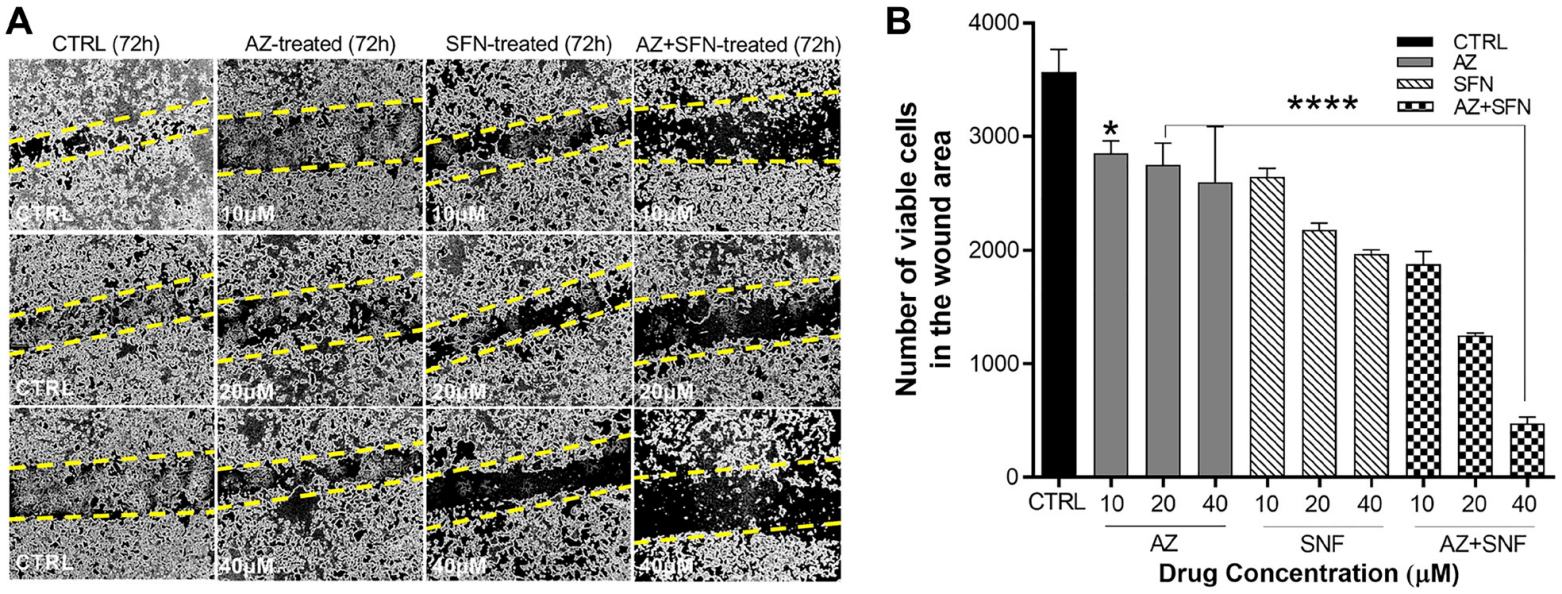
AZ, SFN, and the combination of AZ+SFN dose-dependently block the wound closure ability of typical H727 BC cells using the scratch wound assay. (**A**) AZ, SFN, and the combination of AZ+SFN dose-dependently affect the ability for wound closure of typical H727 BC cells measured over 72 hours compared to untreated control cells using a scratch wound assay. (A) Pictures representative of two separate experiments (*n* = 2) performed in triplicates showing similar results. (**B**) Data from the scratch wound assay show the number of viable cells in the wound closure area ± SEM of three independent experiments performed in triplicates. The data were compared to the untreated control for each drug concentration treatment group by ANOVA using the uncorrected Fisher’s LSD multiple comparisons test with 95% confidence with indicated asterisks for statistical significance. ^*^ *p* ≤ 0.01, ^****^ *p* < 0.0004, *n* = 3. Abbreviations: AZ: acetazolamide; SFN: sulforaphane; BC: bronchial carcinoma.

Patients with bronchial carcinoids present as atypical carcinoids have a substantially lower 5-year survival rate of 25 to 69% due to their greater metastatic and invasive potential [19]. Since atypical H720 BC cells are non-adherent, we investigated the scratch wound closure of the adherent, typical H727 cells at three different concentrations of AZ, SFN, and AZ+SFN (10 μM, 20 μM, and 40 μM) by counting the total number of cells within the wound area. AZ and SFN alone at 10 μM, 20 μM, and 40 μM and their combination significantly inhibited closure of the scratch wound area compared to the untreated control group (Figure 1A and 1B). These findings demonstrate that SFN combined with AZ supports the nutrigenetic therapeutics of SFN in suppressing the migratory/invasive potential of typical bronchial carcinoids.

### In lung bronchial carcinoid (BC) xenografts, AZ, SFN, and AZ+SFN target serotonin (5-HT)-producing tumor cells derived from pulmonary neuroepithelial bodies (NEB)

**Figure 2:**
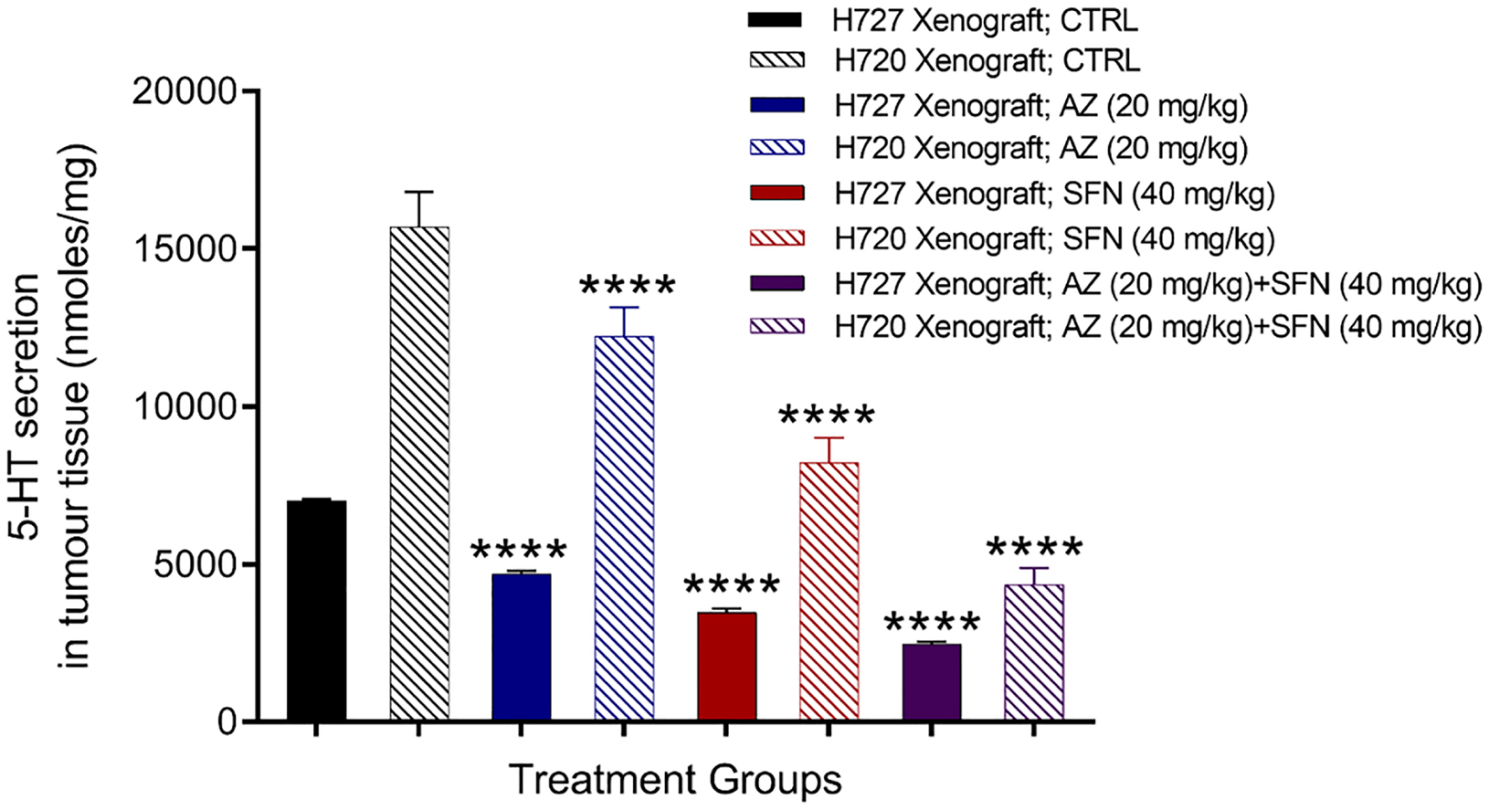
Serotonin 5-HT expression in atypical H720 and typical H727 BC xenografts taken from NOD/SCID mice following treatment with AZ (20 mg/mL), SFN (40 mg/mL), and their combination using the carbon fiber amperometry assay and standard 5-HT calibration curve. Using previous BC H727 (typical) and H720 (atypical) xenografts developed in NOD/SCID mice, treated with AZ (20 mg/kg), SFN (40 mg/kg), and a combination of AZ (20 mg/kg) plus SFN (40 mg/kg), daily for two weeks [3], tumor tissues were dissected. The amount of 5-HT (nmol/mg) in the tissue slices was assessed by amperometry measurements and a standard 5-HT calibration curve. The data are tumor tissue 5-HT secretion as a mean percent of control ± SD of three independent experiments performed in triplicates. The expression of 5-HT was compared to the untreated control for each treatment group by ANOVA using the uncorrected Fisher’s LSD multiple comparisons test with 95% confidence with indicated asterisks for statistical significance. ^****^ *p* ≤ 0.0001, *n* = 3. Abbreviations: AZ: acetazolamide; SFN: sulforaphane; 5-HT: 5-hydroxytryptamine; BC: bronchial carcinoma.

Pulmonary neuroepithelial bodies (NEB) are innervated serotonin (5-HT)-producing cells distributed throughout the airway epithelium. Studies have reported that 5-HT secretion can stimulate cancer progression, including cancer proliferation, migration, and invasion [20, 21]. The data in Figure 1 provide evidence that AZ, SFN, and their combination show dose-dependent reduction in scratch wound closure by the bronchial carcinoid H727 (typical carcinoid) cells. Here, we questioned whether AZ, SFN, and their combination affect 5-HT-producing pulmonary NEB cells in lung bronchial carcinoid xenografts. Using previous BC H727 (typical) and H720 (atypical) xenografts developed in NOD/SCID mice, treated with AZ (20 mg/kg), SFN (40 mg/kg), and a combination of AZ (20 mg/kg) plus SFN (40 mg/kg), daily for two weeks [3], tumor tissues were dissected. The amount of 5-HT (nmol/mg) in the tissue slices was assessed by amperometry measurements and a standard 5-HT calibration curve. It is noteworthy that BC H720 (atypical BC) xenografts expressed higher levels of 5-HT than the BC H727 (typical BC) xenografts (Figure 2). Bronchial carcinoids presenting as typical carcinoids are well-differentiated, rarely metastasize and have a good prognosis with a survival rate of 87 to 100%. However, atypical BC has a substantially lower 5-year survival rate of 25 to 69%, mainly due to their more significant metastatic potential [19]. The data depicted in Figure 2 are consistent with atypical and typical BC properties and characteristics. The atypical BC H720 xenografts expressed 2.2-fold higher levels of 5-HT than the typical BC H727 xenografts. The treatments with AZ and SFN alone significantly reduced the 5-HT expression in both the atypical BC H720 and typical BC H727 xenografts (Figure 2).

### AZ, SFN, and AZ+SFN induce BC cell apoptosis

**Figure 3:**
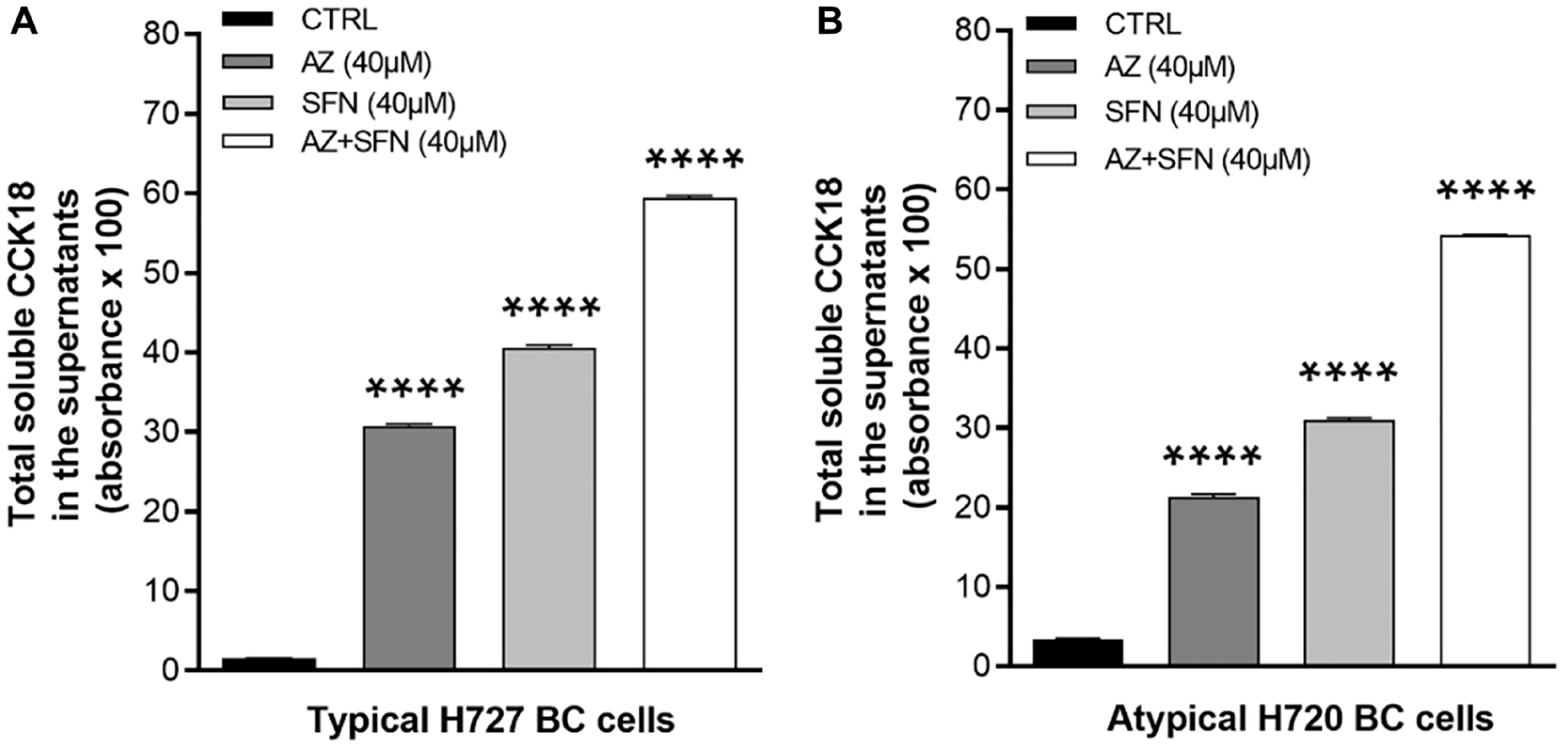
Total soluble caspase-cleaved cytokeratin 18 (CCK18) released from (**A**) H727 and (**B**) H720 dying cells in the supernatants after treatment with AZ (40 μM), SFN (40 μM), and the combination AZ+SFN (40 μM each) for 72 hours using the caspase-cleaved cytokeratin 18 (M65^**®**^, CCK18) CytoDeath ELISA Assay. The data are total CCK18 absorbance as a mean ± SEM of three independent experiments performed in triplicates. The results show a significant progressive increase in CCK-18 from AZ, SFN to the AZ+SFN combination. The expression of CCK18 was compared to the untreated control for each treatment group by ANOVA using the uncorrected Fisher’s LSD multiple comparisons test with 95% confidence with indicated asterisks for statistical significance. ^****^ *p* ≤ 0.0001 *n* = 3. Abbreviations: AZ: acetazolamide; SFN: sulforaphane; CCK18: caspase-cleaved cytokeratin 18; ELISA: enzyme-linked immunosorbent assay; BC: bronchial carcinoma.

Isothiocyanates such as SFN can regulate the expression of p21 and inhibit cell proliferation at the G2-M cell cycle checkpoint [22]. However, many bioactive nutrigenetic compounds can induce the apoptotic pathway, mediated through the mitochondria [23]. We have previously reported that AZ, SFN, and their combination reduced the cell viability of BC cells [3] and induced apoptosis of bladder cancer cells [4]. To determine whether bioactive SFN combined with AZ can induce apoptosis of atypical H720 and typical H727 BC cells, we initially performed the caspase-cleaved cytokeratin 18 (M65, CCK18) CytoDeath ELISA assay. We compared the total CCK18 in the supernatants of H727 (typical) and H720 (atypical) BC cells following 72 hours treatment with AZ (40 μM), SFN (40 μM), and the combination of AZ (40 μM)+SFN (40 μM). In H727 cells, AZ and SFN alone induced CCK18 by 31% and 41%, respectively, while the combination treatment yielded a 60% CCK18 induction compared to the control (^***^*p ≤ 0.0001) (Figure 3A). Similarly, in atypical BC H720 cells, the induction of CCK18 by AZ, SFN, and AZ+SFN was 22%, 32%, and 55%, respectively, compared to the control (^**^ *p* ≤ 0.01, ^***^ *p* ≤ 0.001) (Figure 3B).

**Figure 4:**
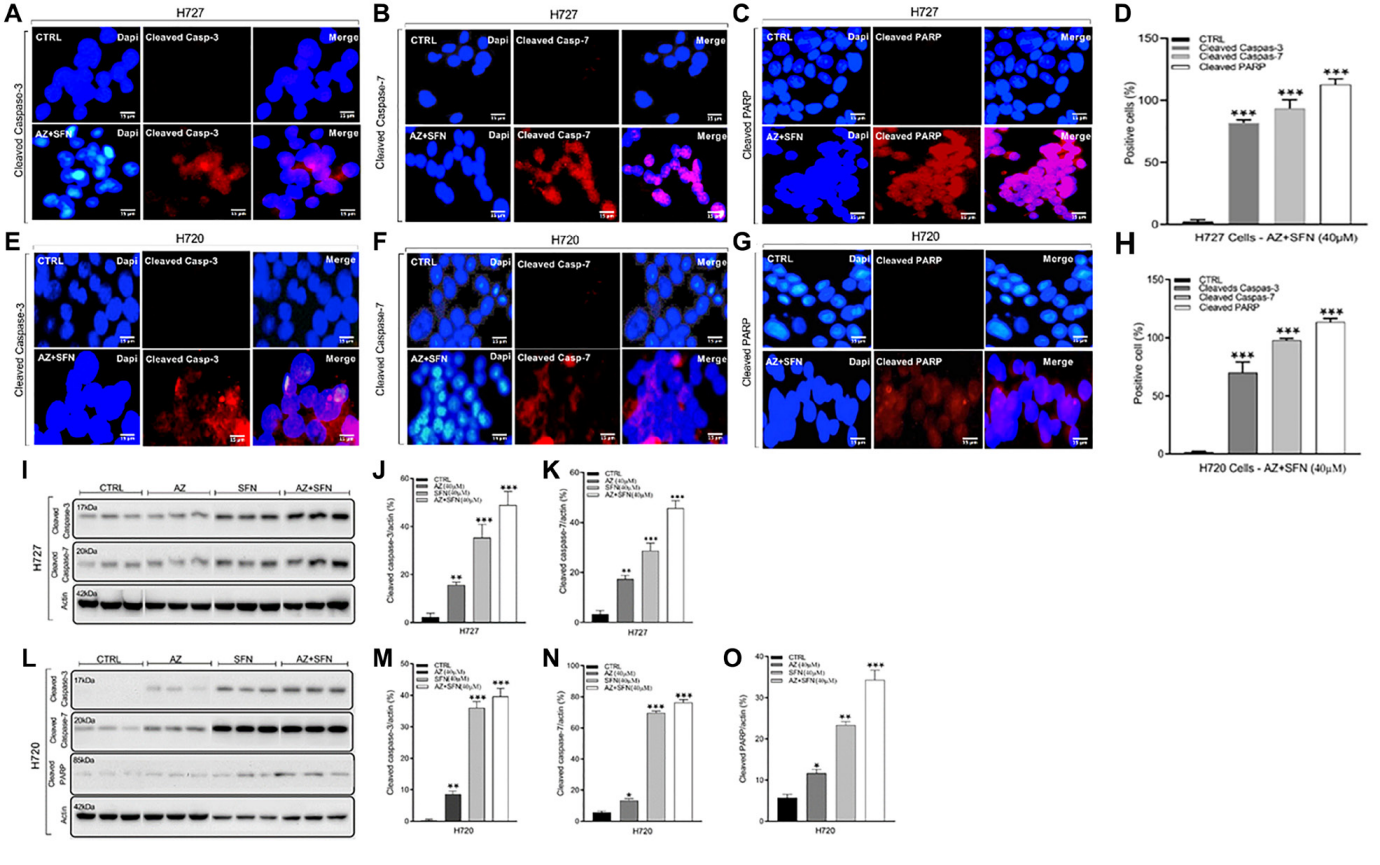
Cleaved caspase-3, cleaved caspase -7, and cleaved PARP expressions in typical H727 (**A**–**D**) and atypical H720 (**E**–**H**) BC cells after AZ (40 μM), SFN (40 μM), and the combination treatment for 72 hours using immunohistochemistry (IHC) and immunofluorescence labeling assays, and Western blot. (D, H) The percent positive cells were quantified using Image J software. (**I**–**O**) Western blot analyses of cell lysates for the expression of cleaved caspase-3, caspase-7, or cleaved PARP. The blots were stripped and reprobed for β actin as a loading control. The data represent one out of three independent experiments showing similar results. The results show a progressive increase in apoptotic proteins from AZ, SFN to AZ+SFN. The expression of cleaved caspase-3, -7, and PARP was compared to the untreated control for each treatment group by ANOVA using the unpaired *t*-test with 95% confidence with indicated asterisks for statistical significance. ^*^ *p* ≤ 0.05, ^**^ *p* ≤ 0.01, ^***^ *p* ≤ 0.001, *n* = 3 independent experiments. Abbreviations: AZ: acetazolamide; SFN: sulforaphane; BC: bronchial carcinoma; PARP: poly [ADP-ribose] polymerase; IF: immunofluorescence.

Bioactive compounds with isothiocyanate properties like SFN have been shown to generally down-regulated anti-apoptotic molecules and upregulate pro-apoptotic molecules [24]. The imbalance between anti-apoptotic and pro-apoptotic proteins can cause the release of cytochrome C from mitochondrial membranes, which in turn forms a complex with caspase-9 and subsequently leads to the activation of caspases-3, –6, and –7 [25]. Also, activated cleaved caspase-3, a cysteine protease, is involved in the early phase of cellular apoptosis and is a crucial regulator of tumor cell repopulation generated from dying cells. Here, we examined cleaved caspase-3, cleaved caspase-7, and cleaved PARP expressions in H727 and H720 cells after AZ, SFN, and the combination treatment for 72 hours with immunohistochemistry (IHC) and immunofluorescence labeling assays to determine whether the bioactive nutrigenetic SFN in combination with AZ can induce mitochondrial early phase of the apoptotic pathway. The data depicted in Figure 4A–4H demonstrate that the treatment regimen significantly induced cleaved caspase-3 (H727: 72%; H720: 70%), cleaved caspase-7 (H727: 89%; H720: 98%), and cleaved PARP (H727: 113%; H720: 115%), compared to control. These results were further confirmed with Western blot (WB) analysis of cell lysates. In typical H727 BC cells, results revealed that although AZ and SFN alone increased the level of cleaved caspase-3 (AZ: 15%; SFN: 35%) and cleaved caspase-7 (AZ: 17%; SFN: 28%), the combination treatment had the highest induction of cleaved caspase-3 (49%) and cleaved caspase-7 (45%), compared to control untreated H727 cells (^*^ *p* ≤ 0.05, ^**^ *p* ≤ 0.01, ^***^ *p* ≤ 0.001) (Figure 4I–4K). Similarly, in atypical H720 BC cells, AZ and SFN alone increased the level of cleaved caspase-3 (AZ: 8%; SFN: 36%) and cleaved caspase-7 (AZ: 13%; SFN: 69%), as well as cleaved PARP (AZ: 11%; SFN: 23%), compared to the untreated controls. Furthermore, SFN in combination with AZ had the highest induction of cleaved caspase-3 (39%), cleaved caspase-7 (76%), and cleaved PARP (34%), compared to control untreated H720 cells (^*^ *p* ≤ 0.05, ^**^ *p* ≤ 0.01, ^***^ *p* ≤ 0.001) (Figure 4L–4O). These results for typical H727 and atypical H720 BC cell lines suggest that the AZ+SFN combination treatment affects a broad range of targets involved in the early phase of cellular apoptosis, thereby upending critical regulators of tumor repopulation generated from the dying cells.

**Figure 5:**
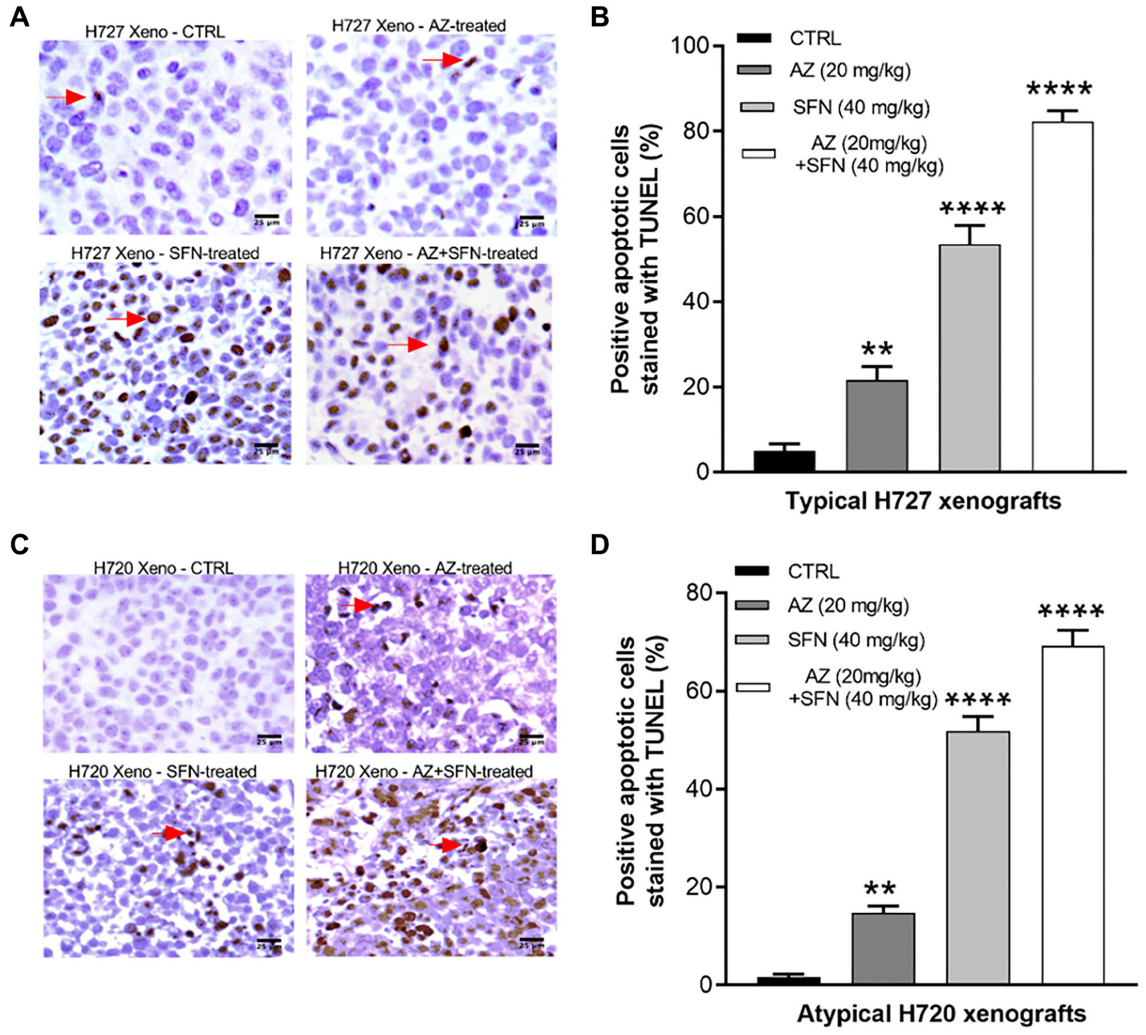
AZ, SFN, and AZ+SFN induce *in vivo* TUNEL apoptosis in BC xenografts. Using previous BC H727 (typical) and H720 (atypical) xenografts developed in NOD/SCID mice, treated with AZ (20 mg/kg), SFN (40 mg/kg), and a combination of AZ (20 mg/kg) plus SFN (40 mg/kg), daily for two weeks [3], tumor tissues were dissected. The amount of apoptosis in the tissue slices was assessed by the TUNEL assay. AZ, SFN, and AZ+SFN increase TUNEL apoptosis levels in (**A**, **B**) H727 cells by 21%, 53%, and 82%, respectively, and (**C**, **D**) H720 cells by 15%, 52%, and 72%, respectively, compared to the untreated control. The expression of TUNEL positive cells was compared to the untreated control for each treatment group by ANOVA using the uncorrected Fisher’s LSD multiple comparisons test with 95% confidence with indicated asterisks for statistical significance. ^**^ *p* ≤ 0.005, ^****^ *p* ≤ 0.0001, *n* = 3 independent experiments. Abbreviations: AZ: acetazolamide; SFN: sulforaphane; BC: bronchial carcinoma; TUNEL: terminal deoxynucleotidyl transferase dUTP nick end labeling.

To further assess late-stage apoptosis, we also examined DNA fragmentation in apoptotic cells by performing terminal deoxynucleotidyl transferase (TdT)-mediated dUTP nick end labeling (TUNEL) assay. Here, we used unstained paraffin section slides obtained from xenografts of H720 and H727 mouse models of BC tumors treated daily for two weeks with AZ, SFN, and their combination. Data demonstrated that AZ, SFN, AZ+SFN increased the number of TUNEL apoptotic cells by 21%, 53%, and 82%, respectively, in H727 xenografts (Figure 5A and 5B) and by 15%, 52%, and 72%, respectively, in H720 xenografts compared to control (^*^ *p* ≤ 0.05, ^**^ *p* ≤ 0.01, ^***^ *p* ≤ 0.001, *n* = 3) (Figure 5C and 5D). These results support the presence of late-stage apoptosis in both H727 and H720 xenografts in the BC mouse model after daily treatments of AZ, SFN, AZ+SFN for two weeks.

### SFN, AZ, and their combination upends hypoxia-induced microenvironment in BC xenografts

Therapy-induced hypoxia can change the tumor microenvironment, contributing to the therapy’s inadequate response. We reported that AZ reduced HIF-1α and tumor-specific, hypoxia-induced upregulation of carbonic anhydrase IX (CAIX) in neuroblastoma cells [26]. CAIX is a complex response to the evolving low oxygen environment in cancer cells. To investigate hypoxia’s role in BC xenografts following treatment with the combination of AZ and SFN, we used the utility of the Hypoxyprobe™ (pimonidazole hydrochloride) immunohistochemical analysis method, which allows for the assessment of hypoxia in different tissues. Pimonidazole is a 2-nitroimidazole that is reductively specifically activated in hypoxic cells, forming stable adducts with thiol groups in proteins, peptides, and amino acids [27]. The amount of pimonidazole detected is directly proportional to hypoxia levels within tumors.

**Figure 6:**
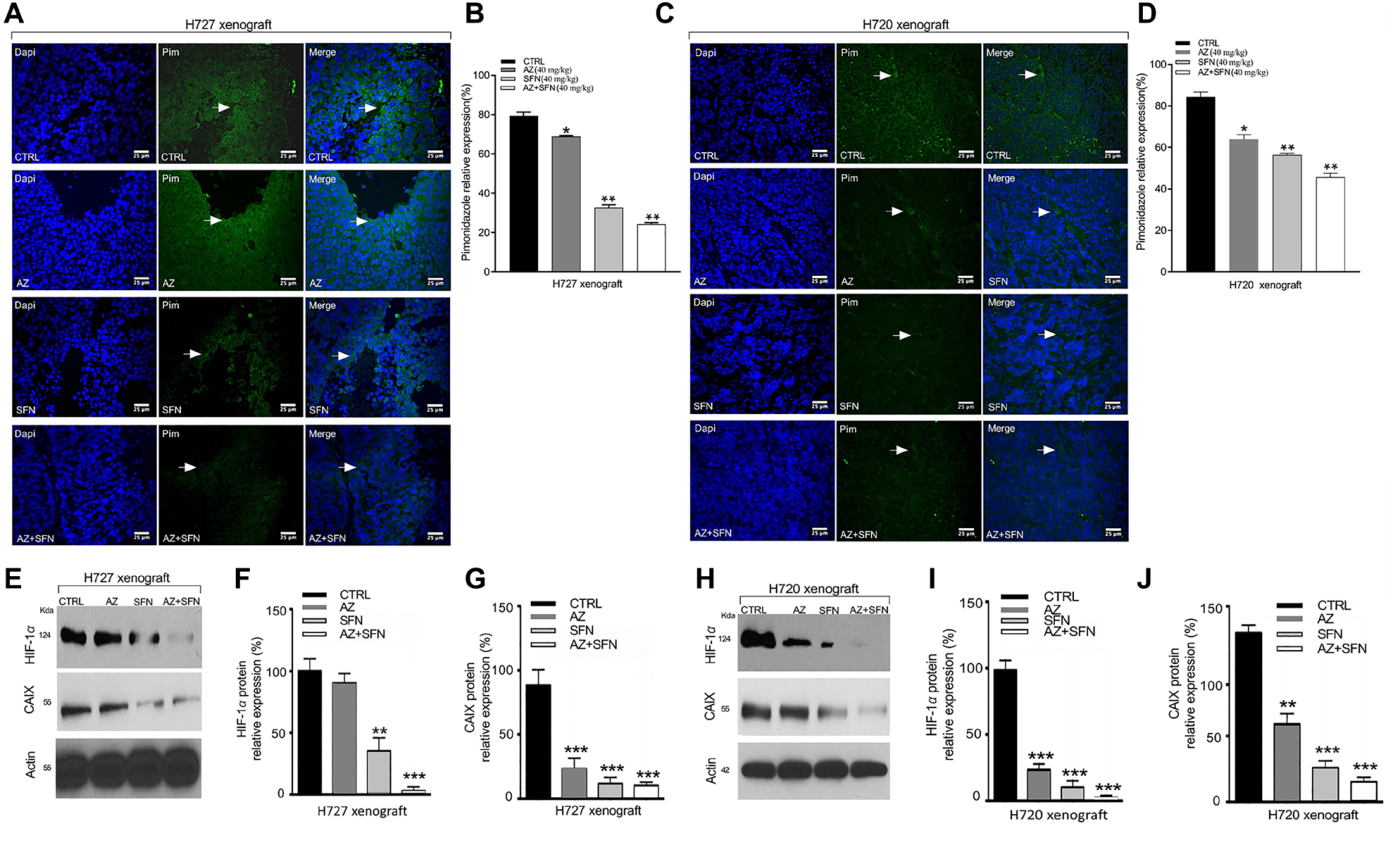
AZ, SFN, and AZ+SFN upend hypoxia microenvironment in H720 and H727 xenografts using Hypoxyprobe™ (pimonidazole hydrochloride) immunohistochemical analysis and western blot of HIF-1α and CAIX in xenograft lysates. Using previous BC H727 (typical) (**A**, **B**) and H720 (atypical) (**C**, **D**) xenografts developed in NOD/SCID mice, the mice were treated with AZ (20 mg/kg), SFN (40 mg/kg), and a combination of AZ (20 mg/kg) plus SFN (40 mg/kg), daily for two weeks [3]. (A–D) Immunofluorescence labeling for pimonidazole (PIM) in tumor sections. Slides were mounted with VectaShield medium with DAPI. (**E**, **H**) Western blot analyses of freshly frozen tumors for the expression of HIF-1α and CAIX. Each lane represents a single tumor lysate. (**F**, **G** and **I**, **J**) The bars in the graphs represent the mean percentage of HIF-1α and CAIX band density ± SEM (error bars) for untreated (CTRL), and treated cohorts for 3–5 replicate measurements. These results confirm that the AZ+SFN combination markedly normalizes the hypoxic state in the xenografts coincident with loss of HIF-1α and CAIX expressions. The data represent one out of three independent experiments showing similar results. ^*^ *p* ≤ 0.05, ^**^ *p* ≤ 0.01, ^***^ *p* ≤ 0.001, *n* = 3. Abbreviations: AZ: acetazolamide; SFN: sulforaphane; BC: bronchial carcinoma; IF: immunofluorescence; HIF-1α: hypoxia-inducible factor 1-alpha; CAIX: Carbonic Anhydrase 9 (CAIX).

We previously used BC xenografts, H727 and H720, in NOD/SCID mice, treated with AZ (20 mg/kg), SFN (40 mg/kg) AZ+SFN combination followed by intraperitoneal injection of pimonidazole according to the kit protocol five hours before euthanasia [3]. In xenografts, pimonidazole expression was assessed by immunofluorescence labeling in sections (5 μm) prepared from new formalin-fixed, paraffin-embedded xenografts tissues. Results revealed that AZ or SFN reduced pimonidazole activity to 78% and 46%, respectively, while combination treatment further reduced expression by 17% in H727 xenograft cells, compared to control (Figure 6A and 6B). Similarly, in H720 xenograft cells, AZ or SFN reduced the expression of pimonidazole activity to 85% and 48%, respectively, while combination treatment further reduced pimonidazole expression by 31%, compared to control (^*^ *p* ≤ 0.05, ^**^ *p* ≤ 0.01, ^***^ *p* ≤ 0.001, *n* = 3) (Figure 6C and 6D). The data indicate that SFN combined with AZ significantly upends the tumor-induced hypoxia microenvironment.

Furthermore, Western blot analyses of xenograft lysates showed that AZ and SFN reduced the expression of HIF-1α by 75% and 90%, respectively, and the expression was further reduced by 98% by a combination of AZ and SFN treatment in typical H727 xenografts, compared to control (Figure 6E and 6F). In H720 xenografts, AZ and SFN alone reduced the expression of HIF-1α by 7% and 60%, respectively, while combination treatment reduced HIF-1α expression by 97% in atypical H720 xenografts, compared to the control (Figure 6H and 6I). AZ, SFN, and AZ+SFN also reduced CAIX expression by 80%, 90%, and 91%, respectively, in H727 xenografts (Figure 6E and 6G) and 40%, 75%, and 80% respectively in H720 xenograft cell respectively, compared to control (^*^ *p* ≤ 0.05, ^**^ *p* ≤ 0.01, ^***^ *p* ≤ 0.001, *n* = 3) (Figure 6H and 6J). The data indicate that the bioactive SFN combined with AZ decreased intra-tumor hypoxia and the subsequent expression of HIF-1α and the downstream effector CAIX involved in in decreasing the level of intra-tumor hypoxia and the downstream effector CAIX involved in low oxygen BC xenografts.

### AZ, SFN, and AZ+SFN upend the PI3K/Akt/mTOR pro-survival pathway of typical H727 and atypical H720 xenografts

**Figure 7:**
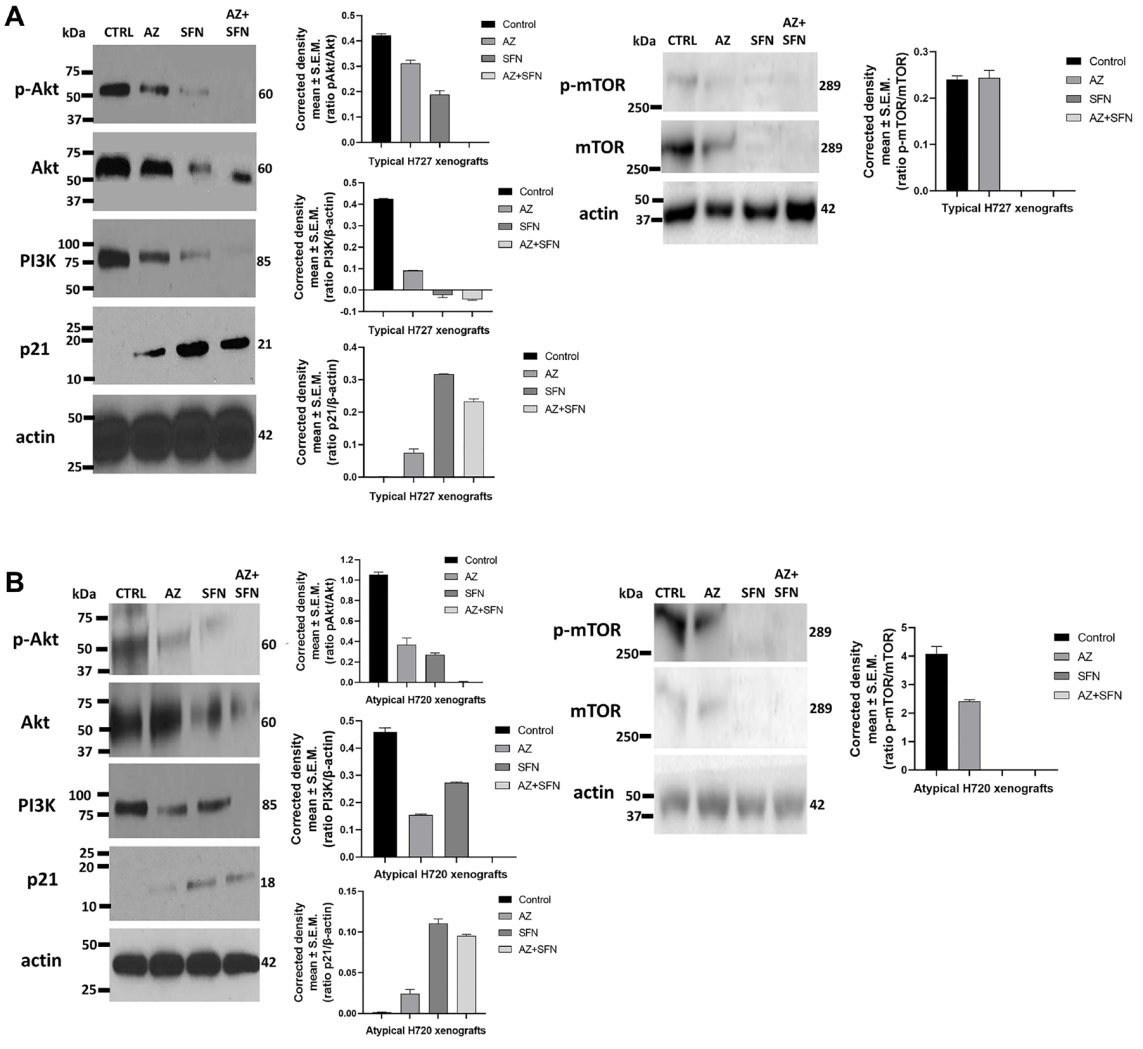
Effect of AZ, SFN, and AZ+SFN on the PI3K/Akt/mTOR signaling pathway in heterotopic xenografts (**A**) typical H727 and (**B**) atypical H-720 tumors growing in NOD/SCID mice. Individual necropsy tumors were taken from untreated control (CTRL) and AZ (20 mg/kg), SFN (40 mg/kg), and a combination of AZ (20 mg/kg) + SFN (40 mg/kg) mice daily treated for two weeks, as previously reported [3]. Each lane represents a single tumor lysate. Quantitative analysis was done by assessing the band’s density corrected for background in each lane using Corel Photo-Paint 8.0 software. Each bar in the graphs represents the mean ratios of pAkt/Akt, PI3K/actin, p21/actin, and p-mTOR/mTOR of band density ± SEM (error bars) for untreated (CTRL) and treated cohorts for 3–5 replicate measurements. Results confirm that the main survival pathways are significantly downregulated by AZ, SFN and especially AZ+SFN. The upregulation of p21 correlates with a significant reduction in tumor growth. The data represent one out of three independent experiments showing similar results. Abbreviations: AZ: acetazolamide; SFN: sulforaphane; BC: bronchial carcinoma; PI3K/Akt/mTOR: phosphoinositide 3-kinases/protein kinase B/mechanistic target of rapamycin; p-Akt: phospho- protein kinase B; p21: cyclin-dependent kinase inhibitor 1; p-mTOR: phospho- mechanistic target of rapamycin.

The bioactive SFN can regulate the expression of p21 and inhibit cell proliferation at the G2-M cell cycle checkpoint [22]. Also, many bioactive compounds can target enzymes through activation of signal transduction pathways such as mitogen-activated protein kinase (MAPK), protein kinase C (PKC), and phosphatidylinositol 3-kinase (PI3K) pathways [28]. In support of this premise, we previously reported that the bioactive SFN combined with AZ upended the PI3K/Akt/mTOR survival signal transduction pathway in bladder tumor cells [4]. In the present study, AZ, SFN, and AZ+SFN markedly reduced the expression ratios of phospho-Akt to Akt and p-mTOR to mTOR as well as the expression of PI3K in typical H727 (Figure 7A) and atypical H720 (Figure 7B) xenografts from NOD/SCID cohort mice treated with AZ (20 mg/kg), SFN (40 mg/kg) and their AZ+SFN combination daily for two weeks as previously reported [3]. It is noteworthy that the AZ alone treatment has a minimum effect on the p-mTOR/mTOR ratio for the typical H727 (Figure 7A) and atypical H720 (Figure 7B) xenografts, while the SFN treatment completely abrogated the p-mTOR expression in both of these xenografts.

Interestingly, SFN alone effectively targeted the PI3K/Akt/mTOR pathway and induced the expression of p21. SFN combined with AZ markedly targeted the PI3K/Akt/mTOR pathway’s inhibitory effect. However, AZ had a minimum effect on the expression of p21 on both typical H727 (Figure 7A) and atypical H720 (Figure 7B) xenografts. The findings suggest that SFN combined with AZ treatment showed a markedly effect on inhibiting the PI3K/Akt/mTOR pro-survival pathways. Also, SFN increased the expression of p21 and inhibiting BC tumor cell proliferation at the G2-M cell cycle checkpoint.

### AZ, SFN, and AZ+SFN target the pro-survival Keap1/Nrf2 pathway of BC cells

Sulforaphane (SFN) is a potential chemopreventive agent that can selectively activate the nuclear transcription factor erythroid 2p45 (NF-E2)-related factor 2 (Nrf2)– Kelch-like ECH-associated protein 1 (Keap1)– antioxidant-response element (ARE) core signaling pathway by inducing *de novo* synthesis of phase II detoxifying or antioxidant genes. Several upstream kinases, including phosphatidylinositol 3-kinase (PI3K), can modulate the Nrf2–Keap1–ARE signaling pathway [28].

**Figure 8:**
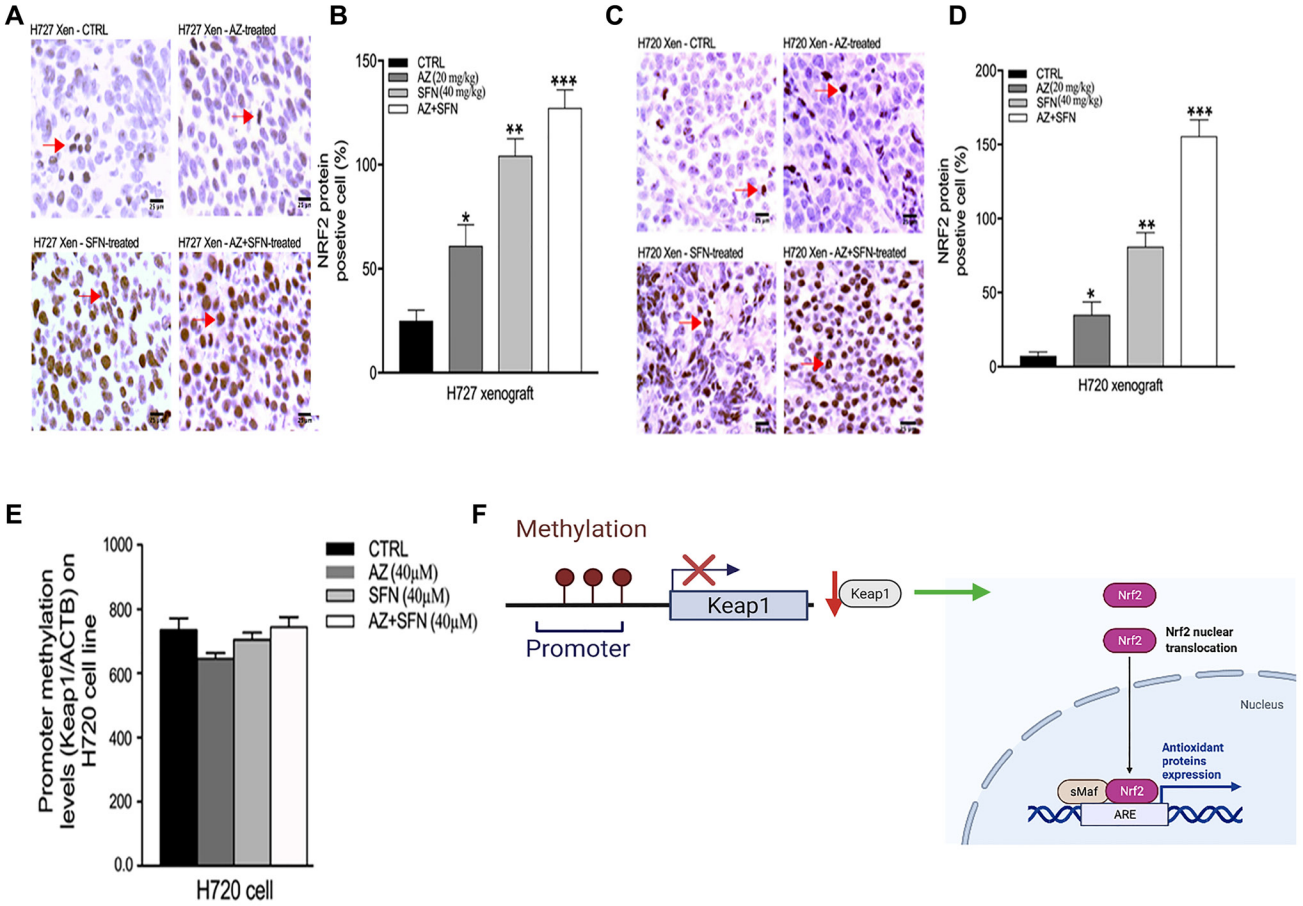
SFN, AZ, and AZ+SFN increase the Nrf2-mediated antioxidant pro-survival pathway in typical H727 (**A**, **B**) and atypical H720 (**C**, **D**) tumors growing in a NOD/SCID mice using immunohistochemistry assay. Individual necropsy tumors were taken from untreated control (CTRL) and AZ (20 mg/kg), SFN (40 mg/kg), and a combination of AZ (20 mg/kg) plus SFN (40 mg/kg) cohorts treated daily for two weeks, as previously reported [3]. The data represent one out of three independent experiments showing similar results. (**E**) RT-PCR data show that Keap1 methylation levels did not change significantly following AZ, SFN, and AZ+SFN treatment in BC H720 cells compared to the untreated control. Nrf2 positive cells’ expression was compared to the untreated control for each treatment group by ANOVA using the unpaired *t*-test with 95% confidence with indicated asterisks for statistical significance. ^*^ *p* ≤ 0.05, ^**^ *p* ≤ 0.01, ^***^ *p* ≤ 0.001, *n* *=* 3. (**F**) Schematic regulation of the promoter and methylation of Keap1; The images were created with https://biorender.com/. Abbreviations: AZ: acetazolamide; SFN: sulforaphane; BC: bronchial carcinoma; Nrf2: nuclear factor erythroid 2–related factor 2.

The data shown in Figure 7 provide evidence that SFN combined with AZ markedly upends the upstream signaling activity of PI3K-Akt-mTOR. Other reports demonstrated that SFN targets the Nrf2 pathway providing antioxidant and chemopreventive activities [29]. Here, we examined if SFN in combination with AZ would specifically affect the Keap1-Nrf2 signaling pathway in a NOD/SCID mouse model of H720 and H727 tumors treated daily for two weeks with AZ (20 mg/kg), SFN (40 mg/kg), and their combination as previously reported by us [3]. Using immunohistochemistry analyses of xenograft tissue sections, the data depicted in Figure 8 reveal that AZ and SFN increased the expression of Nrf2 by 61% and 104%, respectively. Combination treatment further increased expression by 127% in typical H727 xenografts compared to control (Figure 8A and 8B). Similarly, for atypical H720 xenografts, AZ and SFN increased the expression of Nrf2 by 35% and 81%, respectively, while combination treatment further increased expression by 155%, compared to control (^*^ *p* ≤ 0.05, ^**^ *p* ≤ 0.01, ^***^ *p* ≤ 0.001, *n* = 3) (Figure 8C and 8D).

Epigenetic aberrant methylation of the Keap1-Nrf2 axis is considered the most frequent regulation of Keap1 silencing in solid tumors [30, 31]. Muscarella and colleagues have reported on CpG hypermethylation at the P1 promoter region of the Keap1 as one of the epigenetic mechanisms that dysregulate Keap1-Nrf2 cascade in solid tumors, as depicted in Figure 8 [32, 33]. Since aberrant methylation may be one of the mechanisms of altering the Keap1-Nrf2 signaling pathway’s function, we examined methylation of Keap1 using quantitative methylation-specific PCR (qMSP) assay.

The data depicted in Figure 8E reveal that the Keap1 gene did not change its methylation status as compared to controls in the atypical H720 cell line, with no significant changes observed after treatments with AZ (40 μm), SFN (40 μm), and AZ+SFN (40 μm) (Figure 8E). Based on these data, the SFN and AZ treatments were not related to CpG methylation located at the P1 promoter region of the Keap1. These results suggest that the Nrf2 dependent chemopreventive effect of SFN, which increases Nrf2 activity, is likely not associated with the Keap1-Nrf2 axis’s aberrant methylation.

## DISCUSSION

**Figure 9:**
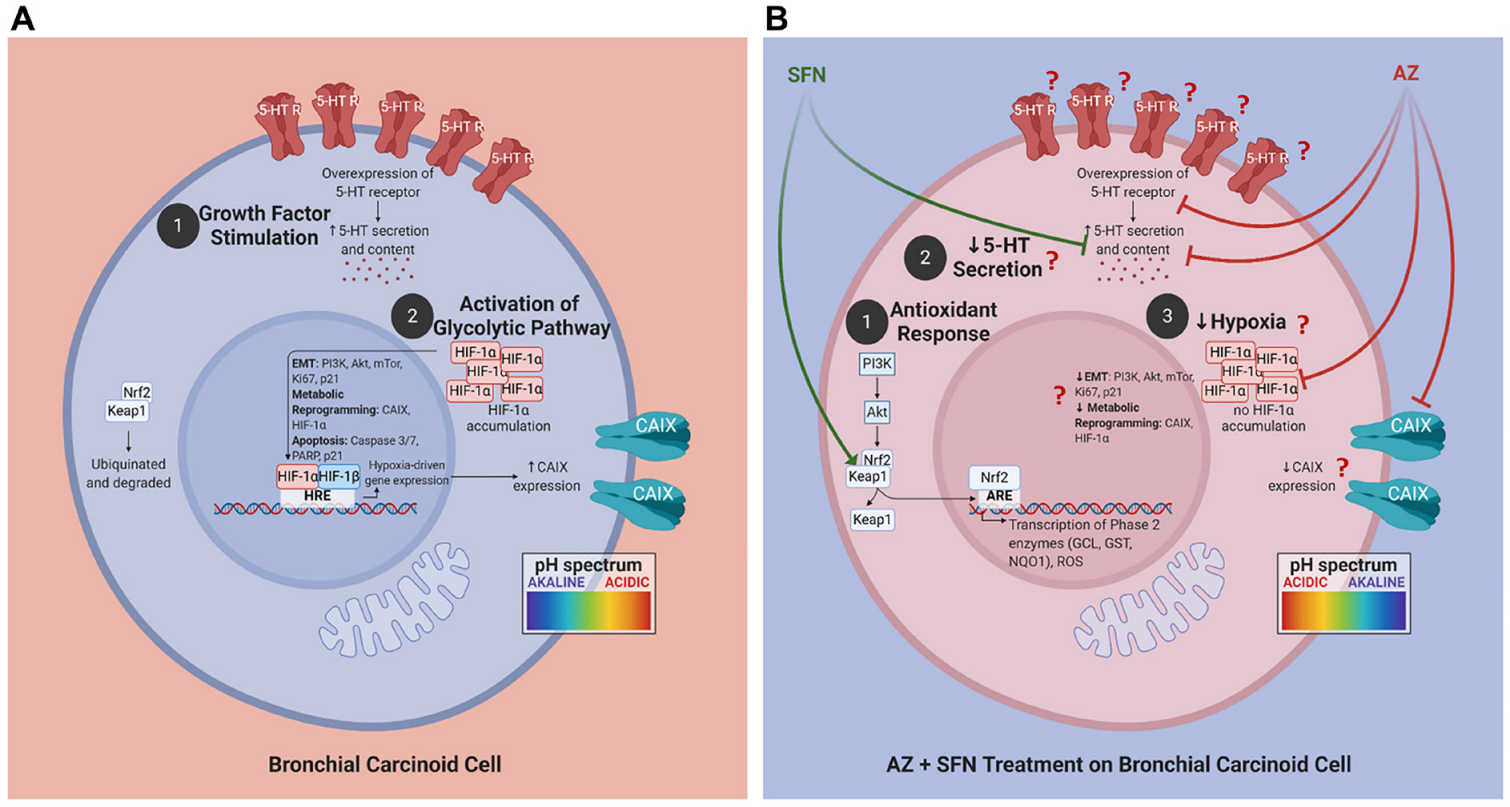
Proposed schema of AZ, SFN, and AZ+SFN targeting the pro-survival pathways in BC. Cells. (**A**) BC cells are in an acidic environment under normal conditions, supporting: (1) growth factor stimulation; and (2) activation of the glycolytic pathway and migratory/invasive potential. (**B**) AZ+SFN treatment works to: (1) modulate the antioxidant response; (2) decrease 5-HT secretion; and (3) decrease hypoxia, making the cell intracellularly more acidic and the extracellular environment more alkaline. The pan-inhibition of these pathways and critical components can ultimately reduce or abrogate BC cells’ tumorigenic potential. The question marks in B require further evidence and study. The images were created with https://biorender.com. Abbreviations: AZ: acetazolamide; SFN: sulforaphane; BC: bronchial carcinoma.

The nutrigenetic therapeutic efficacy of SFN combined with AZ was investigated on bronchial carcinoid cell lines with typical H727 and atypical H720 forms and their xenografts developed in the NOD/SCID mouse model of BC tumors. The research rationale in selecting these BC variant cell lines is that atypical BC tumors have a greater metastatic and invasive potential than the typical BC variant. The combination of SFN and AZ reduced migration of BC in a scratch wound, upended pro-survival hypoxia-mediated pathways resulting in decreased 5-HT secretion, upended the pro-survival PI3K/Akt/mTOR pathway of BC xenografts of H727 and H720 cells, targeted the pro-survival Keap1/Nrf2 pathway, and induced bronchial carcinoid cell apoptosis. For the first time, these findings as depicted in Figure 9 demonstrate that SFN in combination with AZ provides a practical nutrigenetic therapeutic approach in targeting multistage pro-survival pathways in bronchial carcinoids.

Here, we developed a strategic, targeted multimodal approach to take advantage of multiple pro-survival pathways, including tumor cell evasion and chemoresistance. In many cancers, including bronchial carcinoid, aberrations in the PI3K/Akt/mTOR survival pathway are the most common genomic abnormalities. The phytochemical and bioactive SFN has nutrigenomic potential in activating the expression of several cellular protective genes via Nrf2. Nrf2 is primarily related to mechanisms of endogenous cellular defense and survival. Indeed, the data in Figure 8 support the therapeutic potential of SFN in combination with AZ activates Nrf2 in typical and atypical BC xenografts. Also, SFN and AZ have been shown to upend the hypoxia microenvironment in H720 and H727 xenografts that are dysregulated in BC tumors (Figure 6). Here, SFN and AZ increased the expression of Nrf2 by 61% and 104%, respectively, while combination treatment further increased expression by 127% in H727 xenograft tissues compared to untreated control. The nuclear factor Keap1-Nrf2 pathway can promote tumor cell survival in hypoxic conditions [16]. The data in Figure 8E revealed that Keap1 methylation levels did not change significantly following AZ, SFN, and AZ+SFN treatment in BC H720 cells compared to the untreated control.

Nrf2 is an essential downstream effector of the PI3K/Akt/mTOR pathway responsible for modulating oxidative stress by activating phase II detoxifying enzymes and aquaporins [34, 35]. This mechanism is DNA protective in basal conditions, reducing damage from ROS and suppressing tumorigenesis [36]. Keap1 is the primary regulator of Nrf2. Recent evidence suggests that post-initiation dysregulation of the Keap1-Nrf2 system results in constitutive Nrf2 activity responsible for inhibiting apoptosis, metabolic reprogramming, and chemotherapeutic resistance [37]. We previously presented a thorough summary of the current knowledge surrounding the epigenetic mechanisms that dysregulate the Keap1-Nrf2 cascade in solid tumors [33]. Lung, ovarian, gallbladder, and liver cancers are known to have defects in Keap1 that result in Nrf2 over-activation [17]. A loss of function mutation of Keap1 is frequently associated with drug-resistant non-small cell lung cancer [38]. Alternatively, the upregulation of Nrf2 in non-cancerous cells has been shown to induce apoptosis [39]. Figure 8 shows that AZ, SFN, and AZ+SFN do not significantly alter the Keap 1 methylations levels. Sparaneo et al. [33] extensively reviewed the aberrant (hypermethylation) of the Keap1-Nrf2 axis in solid tumors. Hypermethylation of the Keap1 promoter frequently correlates with poor prognosis. Since Nrf2 was markedly upregulated in BC tumor cells after treatments with AZ, SFN and AZ+SFN, the data suggest that the protective function of Nrf2 was disabled in BC.

Studies have shown that AZ and SFN increase Nrf2 activity by suppressing the formation of intestinal polyps in the multiple intestinal neoplasia mouse model with a mutant allele of the murine Apc (adenomatous polyposis coli) locus, encoding a nonsense mutation at codon 850 [33]. In multiple intestinal neoplasia, SFN enhances Nrf2 activity in ovarian carcinoma cells [35]. In recent publications, we reviewed the role of Nrf2 activation in regulating the antioxidant response and tumorigenesis and the effects of single therapy or combination therapy of natural (e.g., SFN) and chemotherapeutic (e.g., doxorubicin) agents on these pathways [40, 41]. In the present study, the treatment of the H727 and H720 cell lines with the combination of AZ+SFN significantly upregulated Nrf2 expression and was associated with increased apoptosis (Figure 8). Although the Nrf2-dependent chemopreventative effects of SFN and other isothiocyanates are well characterized in the literature, increased Nrf2 activity is rarely associated with apoptosis after transformation takes place. It has been hypothesized that perturbations in calcium homeostasis due to Nrf2 modulation of inositol 1,4,5-trisphosphate receptors (IP3Rs) may be responsible for this effect [35].

It should be noted that Keap1 regulates Nrf2 cytoplasmic levels. A wide variety of additional critical cellular proteins, such as pro-apoptotic members of the Bcl-2 family of proteins, regulate cell death and survival [42]. Consequently, low levels of Keap1 induced by epigenetic promoter silencing should enhance apoptosis escape and survival of BC tumor cells via both Nrf2 and Bcl-2 [42]. Thus, Nrf2 may act in both a Keap1-dependent and -independent manner following treatment with AZ, SFN, and AZ+SFN. In the present study, treatment with AZ, a pan-CA inhibitor, suggests that targeting key pro-survival pathways may not significantly involve the PI3K/Akt/mTOR or Nrf2 pathways. However, when combined with SFN, there is a beneficial additive effect on reducing tumorigenic and metastatic potential in BC.

We have previously reported that SFN and AZ combination treatment reduces tumor cell survival compared to each agent alone, both *in vitro* and *in vivo* [3, 4]. Changes in the expression levels of critical molecular markers involved in cell cycle progression, serotonin secretion, survival, and growth pathways were observed following AZ+SFN combination treatment, highlighting multiple pathways that can be targeted by this therapeutic approach. Based on our previous reports showing the therapeutic effect of the AZ+SFN combination [4], we hypothesize that the combination of AZ+SFN exerts its therapeutic efficacy via the PI3k-Akt-mTOR signaling pathways. Both H727 and H720 cells treated with SFN and AZ were associated with induction of apoptosis, upregulation of cell cycle inhibitor protein p21, and downregulation of the PI3K/Akt/mTOR pathway at the molecular level.

The PI3K/Akt/mTOR pathway is a primary target of the SFN+AZ combination therapy [43, 44]. Our previous studies have reported the preferential targeting of the PI3K/Akt/mTOR pathway by the AZ+SFN combination on several bladder cancer pro-survival mechanisms [4]. Although SFN is responsible for a significant proportion of the inhibitory effect, AZ’s addition potentiated this inhibitory effect. Previous reports on other carcinoma cell lines showed treatment with SFN resulted in down-regulation of matrix metalloproteases, MMP-1 and MMP-2, crucial to cancer cell migration and metastasis [43]. This may provide a concordant pathway by which the combination of AZ+SFN reduces tumor wound closure, including migration and proliferation, as depicted in Figure 1A; however, further investigation is warranted to quantify the effects of AZ+SFN on MMPs directly.

LoRusso comprehensively reviewed the molecular alterations in the PI3K-Akt-mTOR pathway that, upon constitutive activation, lead to chemoresistance [45]. A large number of chemotherapeutic agents that can target this pathway was discussed. Tewari et al. [46] more recently reviewed natural products targeting the PI3K-Akt-mTOR pathway activated in cancer cells. It is now established that this pathway is critical for regulating proliferation, transcription, translation, survival, and growth. This signaling axis is subject to multiple stimulatory growth factors and immunomodulators. Interestingly, amongst a large number of possible therapeutic targeting compounds listed, AZ and SFN did not appear. Thus, our studies raise the possibility of effectively targeting this pathway with a clinically amenable combination of AZ+SFN.

The Akt protein kinase has been identified as a critical component of EMT, cancer cell invasion and migration, proliferation, and survival [44]. More specifically, PI3K activation and phosphorylation of the Akt serine/threonine kinase results in downstream activation of DNA repair mechanisms, cell survival mechanisms, and cell cycle progression via Akt inactivation of cell cycle inhibitors, promoting tumor growth and metastasis [5, 47]. Akt also plays an essential role as an anti-apoptotic mediator, where siRNA knockout of the Akt1 and Akt2 isoforms results in increased apoptosis and reduced proliferation in ovarian cancer [48]. In bladder and lung cancer, inhibition of PI3K/Akt/mTOR significantly inhibits migration and tumorigenesis while increasing apoptosis via downregulation of Bcl-2 proteins [49, 50].

Furthermore, mTOR, a downstream component of the PI3K/Akt pathway, has also been reported to be dysregulated in neuroendocrine tumors, playing a critical role in tumorigenesis [51]. As a result, targeting the PI3K/Akt/mTOR pathway has been an area of intense research interest in non-small cell lung cancer [52]. Our finding of reduced mTOR following AZ+SFN treatment is not surprising as SFN has been previously reported to inhibit mTOR in an Nrf2-independent manner; however, this is believed to occur through inhibition of HDAC6, which in turn, decreases the catalytic activity of Akt [53].

Additionally, CAIX overexpression is a hallmark of aggressive and invasive cancer phenotypes associated with a poor prognosis, making transmembrane CAIX an attractive therapeutic target [54]. CAIX facilitates the evasion of apoptosis and survival in hypoxic environments by activating the transcription of a critical set of genes mediated by HIF-1α that assist in internal pH regulation, promote extracellular acidosis conducive to decreased cell adhesion, EMT, and invasion [54–56]. Inhibition of the CAIX catalytic domain has been reported to reduce HeLa cell migration [56]. In this study, AZ+SFN treatment significantly reduced the adherent H727 cells’ migration potential.

Furthermore, CAIX overexpression in hypoxic conditions also promotes tumor angiogenesis, a protective response mediated by HIF-1α-dependent expression of vascular endothelial growth factor (VEGF) and other epigenetic mechanisms [57]. We have previously reported that AZ combined with semisynthetic HDACi, MS275, reduced vascularization in neuroblastoma, *in vitro*, and *in vivo* [26]. Thus, the combination of AZ+SFN may reduce BC survival by reducing vascularization. However, whether this combination can induce BC tumor cell apoptosis or promote hypoxic pro-survival and pro-invasion pathways *in vivo* in patient tumors requires further investigation [58]. CAIX overexpression has also been associated with increased heterogeneity in breast and bladder cancer due to the supportive role of CAIX on the CSC population [4, 12]. The hypoxic tumor microenvironment modulated by CAIX profoundly affects tumor metabolism, resulting in a preferential selection for CSCs. In neuroblastoma, AZ and MS275 reduced the CSC component’s expression *in vitro* and *in vivo* [26]. More recently, we have found that AZ, SFN, and AZ+SFN reduces the expression of stem cell markers (ALDH1, CD44, OCT4, SOX2, and Nanog) in BC cells. [14] Therefore, a reduction in the CSC population may be one mechanism by which AZ+SFN reduced the H727 and H720 cell lines’ tumorigenicity in this study.

CAIX has also been identified as a regulator of 5-HT secretion in NETs due to its cytosolic pH homeostatic role in hypoxic tissues [59]. 5-HT is released by BC and other NETs in response to hypoxic conditions, leading to autocrine growth signaling and subsequent systemic symptoms [3]. We have previously shown that AZ, SFN, and AZ+SFN successfully reduce 5-HT vesicular content in BC. SFN acts synergistically to reduce 5-HT receptor expression, thereby blocking growth signaling in tumor cells [60]. The effectiveness of the combination of AZ and SFN reduces 5-HT content and secretion in H727 and H720 tumor cell xenografts assessed by amperometry (Figure 2).

## MATERIALS AND METHODS

### Cell lines and culture procedures

The bronchial carcinoid cell lines NCI-H727 [H727] (typical carcinoid; ATCC^®^ CRL-5815™) and NCI-H720 [H720] (atypical carcinoid; ATCC^®^ CRL-5838™) used in this study were purchased from the American Type Culture Collection (ATCC). H727 and H720 cell lines were maintained in RPMI-1640 medium (Sigma-Aldrich Canada Inc., Oakville, ON, Canada) containing 10% fetal bovine serum and 0.5% penicillin-streptomycin. The cell lines were routinely monitored for mycoplasma using immunofluorescence detection and the maintenance of the characteristic phenotypes described by ATCC. Cultured cells were incubated in a 5% CO_2_ incubator at 37°C. Cells were passaged once they reached ~80% confluence (approximately every 4–5 days) using Trypsin [14].

### Reagents

Acetazolamide (AZ), dimethyl sulfoxide (DMSO), serotonin hydrochloride (5-HT), D4-serotonin, 5-Hydroxyindole-3-acetic acid (5-HIAA), and trans-2- phenylcyclopropylamine hydrochloride were obtained from Sigma-Aldrich (Oakville, ON, Canada). Sulforaphane was purchased from LKT Laboratories (St. Paul, MN, USA). Culture medium components RPMI-1640, fetal bovine serum (FBS), and penicillin-streptomycin were purchased from Wisent (Saint-Jean-Baptiste, QC, Canada). The heterophilic blocking reagent (HBR) was purchased from Scantibodies (Santee, CA, USA). The cleaved caspase-3, cleaved caspase-7, cleaved PARP, and AlexaFlour antibodies were purchased from Cell Signaling Technology (Toronto, ON, Canada). Pimonidazole and VectaShield were purchased from Burlingame, CA, USA. The phospho-Akt1, Akt1, HIF-1α, PI3K, p21, and CAIX antibodies were purchased from Abcam (Cambridge, MA, USA). The phospho-mTOR, mTOR, and actin antibodies were provided by Dr. Lucia Anna Muscarella’s lab (IRCCS Casa Sollievo della Sofferenza, Italy). The *in-situ* cell death detection kit and DNaseI were purchased from Roche (Indianapolis, IN, USA). The carbon fiber electrode was purchased from Dagan (Izumi, Higashi-Ku, NA, Japan). Axopatch 200B amplifier and Digidata 1440A were purchased from Axon Instruments (Nature Trait LLC, Lewes, DE, USA). EpiTect Bisulfite kit was purchased from Qiagen (Redwood City, CA, USA). The enzymatically methylated human genomic DNA was purchased from CpGenomeTM Universal Methylated DNA (Millipore, MA, USA). DAB solution was purchased from Invitrogen (Grand Island, NY, USA).

### H727 and H720 xenograft tissues from NOD/SCID mouse model of bronchial carcinoid

H727 and H720 xenograft tissue developed in NOD/SCID mice were taken from our previously reported study [3] and used as new samples in this present study. Briefly, H727 (typical carcinoid) and H720 (atypical carcinoid) cells (2 × 10^6^) were subcutaneously injected into the inguinal fat pad of NOD/SCID mice. When the tumors attained a diameter of 0.5 cm, the mice were randomized into four groups (5 mice per group). The control (untreated) and treatment groups received intraperitoneal injections of vehicle (PBS) or AZ (20 mg/kg), SFN (40 mg/kg), and a combination of AZ (20 mg/kg) + SFN (40 mg/kg), respectively, every day for two weeks. The animals were euthanized when tumor sizes exceeded 2 cm^2^ in diameter or animals showed signs of morbidity. Four-to-six-week-old female NOD/SCID mice were obtained from the animal facility at The Hospital for Sick Children (SickKids). They were used accordingly within the Lab Animal Services guidelines. The previous study protocols for animal experimentation were approved by the Animal Safety Committee, SickKids Research Institute, Toronto, Ontario, Canada [3].

### Scratch wound assay

The H727 (typical carcinoid) adherent BC cell line was seeded on glass coverslips at a density of 1×10^5^ cells/well (48-well plate) in a 500 μL culture medium and allowed to adhere overnight as previously reported by us [26]. A straight line was drawn with a permanent marker on each well’s bottom. When cells reached 90% confluence, the cell monolayer was scored with a 200 μL pipette tip along with the marker guide. Non-adherent cells were removed with a medium wash, and a fresh medium containing AZ, SFN, and AZ+SFN (10 μM, 20 μM, and 40 μM) was added to the culture. After completing the experiment, cells were fixed in 4% paraformaldehyde with 1xPBS wash before and after fixation and stained with 1% crystal violet in 20% methanol. Phase-contrast light microscopy (4× magnification) was used to image cells at the 72-hour interval. The wound site’s cell migration area was quantified for live and dead cells utilizing the Image J program. Three independent experiments were conducted in triplicate.

### Caspase-cleaved cytokeratin 18 (M65^®^, CCK18) CytoDeath ELISA assay

In the M65^®^ ELISA, the heterophilic blocking reagent (HBR) was used with this assay, as previously described by Olofsson [61]. The HBR is a specific binder for human heterophilic antibodies, blocking by a steric hindrance to prevent human heterophilic antibodies’ interference in immunoassays. Briefly, HBR reagent was added directly to the diluted conjugate buffer, and the assay procedure was continued as described in the kit datasheet. The M65^®^ ELISA measures soluble caspase-cleaved cytokeratin 18 (CCK18) released from dying cells. They used to measure the overall cell death due to apoptosis and necrosis of cells’ total degree of cancer cell death. The M65^®^ ELISA assay here measured the total soluble CCK18 in the supernatants. The SpectroMax M5 microplate reader was used to determine the signal’s intensity by measuring the absorbance at 450 nm.

### Apoptosis

The cells were cultured on glass coverslips until 75% confluent, as previously described [26]. Cells were treated for 72 hours with AZ (40 μM), SFN (40 μM), and the combination of AZ (40 μM)+SFN (40 μM). Cells were treated for 72 hours with AZ (40 μM), SFN (40 μM), and the combination of AZ (40 μM)+SFN (40 μM). Cells were fixed and permeabilized in 80% cold methanol for 10 minutes on ice, washed three times in cold-PBS and incubated with primary antibodies against cleaved caspase-3 (Asp175, 1:400), cleaved caspase-7 (Asp198, 1:1600), and cleaved PARP (Asp214, 1:400) (Cell Signaling Technology, New England Biolabs, Ltd., Whitby, Ontario, Canada). Cells were incubated in the appropriate AlexaFluor secondary antibodies (1:5000) and mounted in PBS/glycerol with DAPI solution for visualization. The percentage of positive cells was measured using the formula [X (6 low power fields of positive staining)/Y (total count per 6 fields)] × 100.

### Hypoxia

The immunofluorescence labeling assay was performed on new tumor tissues obtained from a previous study [3], having received pimonidazole, fixed and paraffin-embedded. Tissue sections were deparaffinized through xylene and graded alcohols into water. Sections were antigen retrieved in 10 mM sodium citrate buffer (pH 6.0) by pressure heating in a microwave oven for 10 minutes. After cooling to room temperature (20 minutes), non-specific binding was blocked by incubation in 4% BSA/PBS for 10 minutes. Sections were subsequently incubated overnight at 4°C with anti-pimonidazole (MAb1, 1:50) (Hypoxyprobe, Inc.121 Middlesex Turnpike, Burlington, MA, USA) followed by secondary antibody conjugated with FITC. Slides were washed 3× in PBS for 10 minutes and mounted with VectaShield mounting medium with DAPI (MJS BioLynx Inc., Brockville, ON Canada).

### Western blotting

Western blotting was performed as previously reported and described by us [26]. Briefly, cells were lysed with RIPA extraction buffer (MBiotech, Seoul, Korea) supplemented with a Complete Mini protease inhibitor tablet (Roche, Indianapolis, IN, USA). 100 μg of protein was loaded and the primary antibodies and dilutions used were as follows: anti-phospho-Akt1 (Ser473,1:400), anti-Akt1 (1:200), anti-HIF-1α (EP1215Y, 1:2000), PI3K (M253, 1:1000), anti-p21 (EPR18021, 1:1000), anti-CAIX (1:300), anti-p-mTOR (Ser2448, 1:1000), anti-mTOR (Ser2448, 1;1000), and anti-actin (1:5000). Secondary horseradish peroxidase-conjugated antibodies (Jackson Immunoresearch, West Grove, PA, USA) were used at a dilution of 1/6000. The signal was detected with the Super Signal chemiluminescence detection system (Pierce Biotechnology, Rockford, IL, USA). Quantification of bands was done by digitized densitometry.

Freshly frozen tumors were thawed on ice and lysed in lysis buffer containing proteinase and phosphatase inhibitors. The tumor lysates were resolved by SDS-PAGE. The blot was probed with phospho-Akt, Akt, PI3K, p21, phospho-mTOR, and mTOR primary antibodies followed by horseradish peroxidase-labeled goat anti-rabbit antibody and Super Signal chemiluminescence detection system. After development, blots were stripped and reprobed with rabbit anti-β actin as a loading control. Quantitative analysis was done by assessing the density of a band corrected for background in each lane using Corel Photo-Paint 8.0 software. Each bar in the graphs represents the mean ratio of protein to β-actin of band density ± SE (error bars) for 5–10 replicate measurements. The data are a representation of one of three independent experiments showing similar results.

### TUNEL assay

TUNEL assay was performed on previously obtained new tumor sections (5 μm) prepared from formalin-fixed, paraffin-embedded xenograft tumors [3] with the *In Situ* Cell Death Detection Kit (Roche, Indianapolis, IN, USA). Briefly, H727 and H720 cells (2 × 10^6^) were injected into the subcutaneous inguinal fat pad of NOD/SCID mice. When the tumors attained a diameter of 0.5 cm, the mice were randomized into four groups (5 mice per group). The control (untreated) and treatment groups received intraperitoneal injections of vehicle (PBS) or AZ (20 mg/kg), SFN (40 mg/kg), and a combination of AZ (20 mg/kg) + SFN (40 mg/kg), respectively, every day for two weeks [3]. The assay was performed using the kit protocol, and as a positive control, the tissue sections were treated with 500 units/ml DNase I before adding the TUNEL reaction buffer. The peroxidase reaction was performed with a stable DAB solution. The Vector DAB substrate (3,3′-diaminobenzidine) produces a brown reaction product in peroxidase (HRP) enzyme presence. Sections were hematoxylin counterstained and examined under light microscopy. The percentage of positive cells was measured using the formula [X (6 low power fields of positive staining)/Y (total count per 6 fields)] × 100. The number of positive TUNEL cells was also examined by Image J software and compared with control.

### Immunohistochemistry

Standard immunohistochemistry (IHC) protocol was performed using new tumor paraffin sections as previously described [3]. Approximately 7 μm paraffin sections were deparaffinized in xylene and rehydrated through a graded series of alcohols into the buffer. Further processing was performed to block endogenous peroxidase activity and antigen retrieval. After incubation in primary antibodies, washes, and the tissues were stained with a secondary antibody broad-spectrum poly horseradish peroxidase following DAB reaction, or secondary antibody conjugated Alexa Fluor 594 and DAB. The percent positive cells were calculated using the formula [X (6 low power fields of positive staining)/Y (total count per 6 fields) × 100]. The level of IHC positive cells was determined and quantified using Image J software.

### Measurement of serotonin 5-HT concentration in tumor tissue by carbon fiber amperometry assay

Carbon fiber amperometry was used to assess tissue 5-HT concentrations of NEB cells from BC tumor slices, as described by Livermore [62]. Pulmonary neuroepithelial bodies (NEB) are innervated serotonin (5-HT)-producing cells distributed throughout the airway epithelium. Briefly, tissue slices were perfused under gravity at ~2 mL per min on the stage of an inverted microscope (Optiphot-2UD, Nikon, Tokyo, Japan). A carbon fiber electrode (ProCFE, Dagan, Izumi, Higashi-Ku, NA, Japan) polarized to +800 mV was placed near the cell membrane. An Axopatch 200B amplifier and Digidata 1440A (Axon Instruments, Nature Trait LLC, Lewes, DE, USA) pClamp ten was used to record currents. Quantal events were detected as transient positive-going current spikes with exponential decays. Secretion rate was determined as the quantal charge product (proportional to the number of molecules oxidized) and frequency of quantal events.

### Quantitative methylation-specific PCR (qMSP)

According to the manufacturer’s protocol, sodium bisulfite modification and DNA purification from cell lines were performed using the EpiTect Bisulfite kit (Qiagen, Redwood City, CA, USA). Bisulfite-converted genomic DNA was amplified using the qMSP primers/probe set previously described [63]. 10-fold dilutions for four points (0.05–50 ng) of enzymatically methylated human genomic DNA were constructed with a calibration curve for ACTB (reference) and *Keap1* (target) genes. qMSP reactions were performed on the ABI PRISM™ 7900HT Sequence Detection System and analyzed using the SDS 2.4 software (Thermo Fisher, San Francisco, CA, USA).

### Statistical analysis

Data represented as mean ± standard error of the mean (SEM) or standard deviation (SD) from three or more repeats of each experiment performed in triplicate. GraphPad Prism software was used to perform statistical analyses. The Student’s *t*-test (unpaired) or One-way Analysis of Variance (ANOVA) with *p* < 0.05 were used to compare the results as statistically significant (^*^ *p* ≤ 0.05, ^**^ *p* ≤ 0.01, and ^***^ *p* ≤ 0.001).

## CONCLUSIONS

The present study has shown that the combination of AZ and SFN at clinically significant doses is more effective in abrogating BC survival, metastasis and inducing apoptosis than the drugs alone. The therapeutic potentiation from these drugs acts on multiple hypoxia-mediated pro-survival pathways in BC. Notably, SFN downregulation of the PI3K/Akt/mTOR pathway and downstream effectors promoting cell survival is mediated by the simultaneous targeting of CA-dependent hypoxia-mediated pathways by AZ. The perturbation of pH homeostasis plays a crucial role in the efficacy of this combination therapy. It leads to reduced angiogenesis, invasion, and potentially, the preferential apoptosis of the CSC population. From a clinical perspective, reducing the production and secretion of 5-HT should ameliorate the severe carcinoid syndrome associated with BC, thereby improving patient clinical outcomes. In Figure 9, we summarize the observations of AZ, SFN, and the combination of AZ and SFN effects in BC, demonstrating how the simultaneous multi-targeting of key pro-survival pathways (hypoxia, antioxidant, and PI3K/Akt/mTOR) and pH homeostasis could represent a promising therapeutic regimen for BC.

## Abbreviations

5-HT: 5-hydroxytryptamine
AC: typical carcinoid
AZ: Acetazolamide
BC: Bronchial carcinoma
CA: Carbonic anhydrase
CAIX: Carbonic anhydrase IX
CSC: cancer stem cell
E.M.T.: Epithelial-to-mesenchymal transition
HBR: Heterophilic blocking reagent
HIF-1α: Hypoxia-inducible factor-1α
ITC: Isothiocyanate
NET: Neuroendocrine tumor
NF-κB: Nuclear factor-kappa B transcription factor
Keap1-Nrf2: Nuclear factor (erythroid-derived 2)-like 2-kelch-like ECH-associated protein 1
PI3K-Akt: Phosphatidylinositol 3-kinase-Akt
ROS: Reactive oxygen species
SFN: Sulforaphane
TC: typical carcinoid
TUNEL: terminal deoxynucleotidyl transferase (TdT)-mediated dUTP nick end labeling
VEGF: Vascular endothelial growth factor

## Notes

**Editorial Note**

This paper has been accepted based in part on peer-review conducted by another journal and the authors’ response and revisions as well as expedited peer-review in Oncotarget.
