# Correction: Next-generation multimodality of nutrigenomic cancer therapy: sulforaphane in combination with acetazolamide actively target bronchial carcinoid cancer in disabling the PI3K/Akt/mTOR survival pathway and inducing apoptosis

**DOI:** 10.18632/oncotarget.28104

**Published:** 2022-08-04

**Authors:** Reza Bayat Mokhtari, Bessi Qorri, Narges Baluch, Angelo Sparaneo, Federico Pio Fabrizio, Lucia Anna Muscarella, Albina Tyker, Sushil Kumar, Hai-Ling Margaret Cheng, Myron R. Szewczuk, Bikul Das, Herman Yeger

**Affiliations:** ^1^Program in Developmental and Stem Cell Biology, The Hospital for Sick Children, Toronto, Ontario, Canada; ^2^Department of Experimental Therapeutics, Thoreau Laboratory for Global Health, M2D2, University of Massachusetts, Lowell, MA, USA; ^3^Department of Biomedical and Molecular Sciences, Queen’s University, Kingston, Ontario, Canada; ^4^Department of Immunology and Allergy, The Hospital for Sick Children, Toronto, Ontario, Canada; ^5^Laboratory of Oncology, IRCCS Casa Sollievo della Sofferenza, San Giovanni Rotondo FG, Italy; ^6^Department of Internal Medicine, University of Chicago, Chicago, IL, USA; ^7^Q.P.S. Holdings LLC, Pencader Corporate Center, Newark, DE, USA; ^8^Institute of Biomedical Engineering, The Edward S. Rogers Sr. Department of Electrical & Computer Engineering, University of Toronto, Toronto, Canada; ^9^Department of Cancer and Stem Cell Biology, KaviKrishna Laboratory, Guwahati Biotech Park, Indian Institute of Technology, Guwahati, Assam, India; ^10^Department of Immunology and Infectious Diseases, Forsyth Institute, Cambridge, MA, USA

**This article has been corrected:** Due to errors in figure preparation, the AZ-treated (72 hr) images (row 1, panel 2), is an accidental duplicate of the ‘CTRL’ image in row 3, panel 1 of Figure 1A. The corrected Figure 1A, obtained using the original data, is shown below. The authors declare that these corrections do not change the results or conclusions of this paper.

Original article: Oncotarget. 2021; 12:1470–1489. 1470-1489. https://doi.org/10.18632/oncotarget.28011

**Figure 1:**
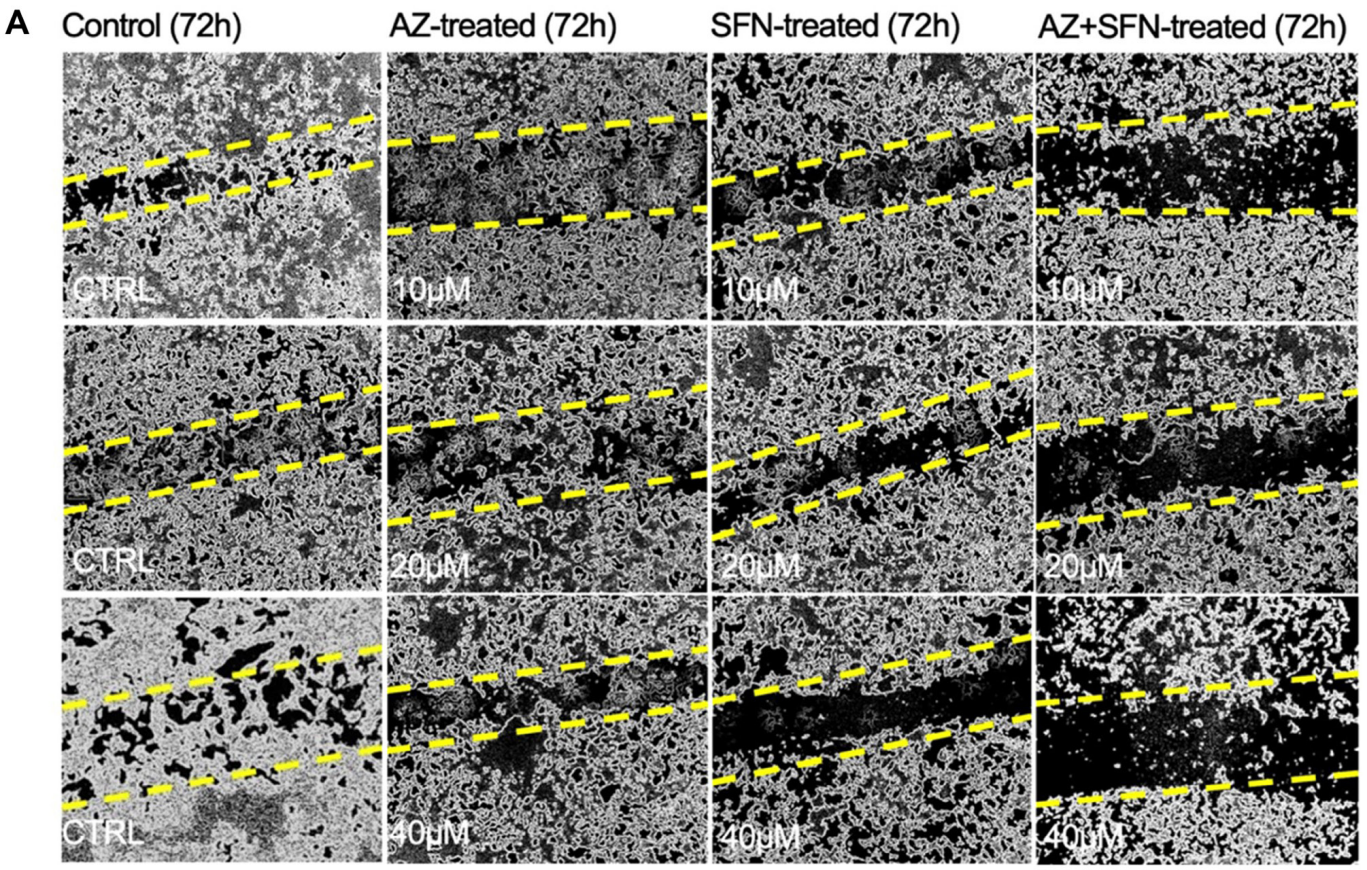
(**A**) AZ, SFN, and the combination of AZ+SFN dose-dependently affect the ability for wound closure of typical H727 BC cells measured over 72 hours compared to untreated control cells using a scratch wound assay. (A) Pictures representative of two separate experiments (*n* = 2) performed in triplicates showing similar results.

